# P-1750. Assessment of Antimicrobial Stewardship Guided CYP2C19 Pharmacogenomic Voriconazole Dosing: Practicalities and Impact on Clinical Management

**DOI:** 10.1093/ofid/ofae631.1913

**Published:** 2025-01-29

**Authors:** Julian Lindsay, Elizabeth M Krantz, Allison Thibodeau, Frank Tverdek, Zahra Escobar, Avadhut Joshi, Rosa Yeh, Michael J Boeckh, Steven A Pergam, Catherine Liu

**Affiliations:** Fred Hutchinson Cancer Center, Seattle, Washington; Fred Hutch Cancer Center, Seattle, Washington; Fred Hutch Cancer Center, Seattle, Washington; Fred Hutchinson Cancer Center, Seattle, Washington; Fred Hutchinson Cancer Center, Seattle, Washington; Fred Hutchinson Cancer Center, Seattle, Washington; Fred Hutchinson Cancer Center, Seattle, Washington; Fred Hutchinson Cancer Center, Seattle, Washington; Fred Hutchinson Cancer Center; University of Washington, Seattle, WA; Fred Hutchinson Cancer Center, Seattle, Washington

## Abstract

**Background:**

Pharmacogenomic testing of CYP2C19 is a potential tool to optimize dosing of voriconazole (VCZ), a drug with a narrow therapeutic index and interpatient variability. An onsite CYP2C19 assay was developed to improve the accessibility and turnaround time of results. In this pilot study, we evaluated the utility and clinical impact of antimicrobial stewardship (AMS)-guided CYP2C19 testing.

**Figure:**
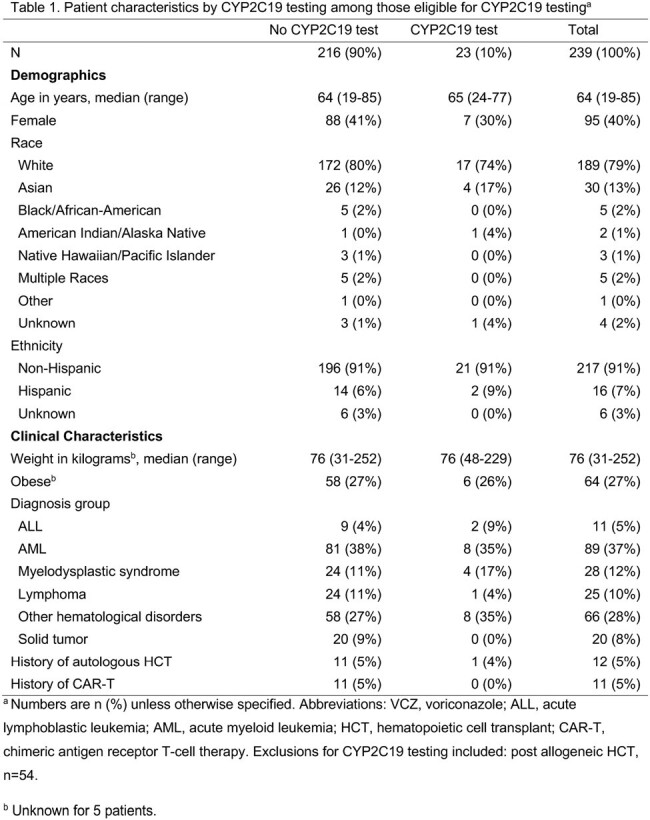

**Methods:**

Starting 5/1/2022, the AMS team provided recommendations regarding CYP2C19 testing and dosing for selected patients receiving VCZ at our comprehensive cancer center. CYP2C19 testing was performed using RT-PCR with allele-specific probes for *2, *3, *6, *8, *9, *10, and *17. We conducted a retrospective chart review of all adult cancer patients with a VCZ order between 5/1/2022 and 10/31/2023 and captured clinical characteristics, result turnaround time, VCZ dose adjustment and therapeutic levels.

**Figure:**
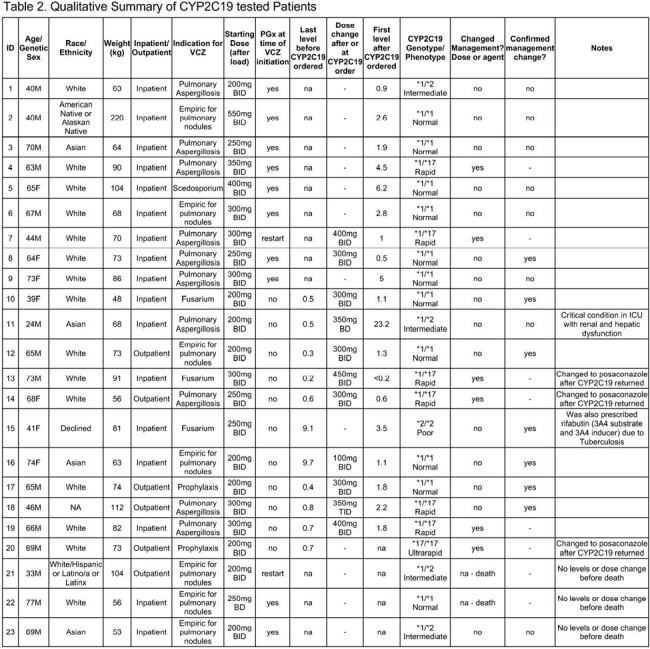

**Results:**

Among 239 patients prescribed VCZ that were eligible for CYP2C19 testing, 23 (10%) were tested for CYP2C19 (Table 1), 10 at time of VCZ initiation, 2 when restarting a VCZ course, and 11 with ongoing VCZ following sub/supratherapeutic levels (Table 2, Figure 1). In 6/23 (26%) patients, VCZ management was impacted by guiding dose change or alternative antifungal based on CYP2C19 genotype. In 7/23 (30%) a dose change was made while the genotype was pending, with the results confirming management. There was no impact on management in 8/23 (35%), and 2/23 (9%) patients died before a dose change could be made. Of 11 patients with initial sub/supratherapeutic VCZ levels before testing, 5/11 were CYP2C19 ultra/rapid metabolizers; 10/11 either changed or confirmed management based on CYP2C19 genotype. Of these, 7/10 had a therapeutic level after first dose adjustment, while 3/10 changed antifungal. The median time from CYP2C19 order to results was 4.3 days (interquartile range [IQR] 3.0-5.5), (Figure 2).

**Figure 1.**
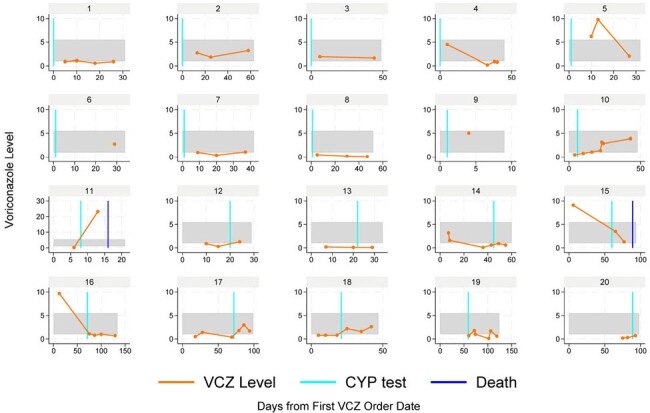
Voriconazole levels among 20/23 patients with CYP2C19 testing and VCZ levels. Gray horizontal band represents the therapeutic range (1.0-5.0 mg/L). Levels <0.2 mg/L were assigned a value of 0.1.

**Conclusion:**

AMS-guided onsite CYP2C19 testing impacted clinical management of VCZ dosing or selection of alternative antifungals. While our data suggest its utility in patients with sub/supratherapeutic levels, further research is needed to evaluate its role in guiding initial VCZ dosing in high-risk patients.

**Figure 2.**
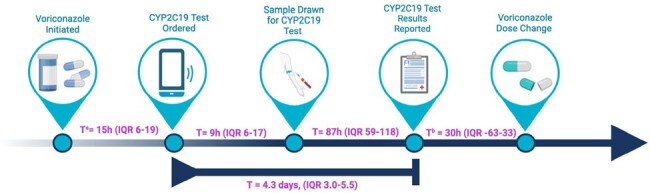
CYP2C19 guided voriconazole dose adjustment timeline with the median time and interquartile range [IQR] in hours (h) or days between steps.

(a) In new/restart VCZ orders only, n=12; (b) In those with dose changes based on, or pending CYP2C19 results, n=9.

**Disclosures:**

**Julian Lindsay, BPharm, MClinPharm, PhD**, Amgen: Advisor/Consultant|BMS: Advisor/Consultant|Mayne: Advisor/Consultant|Merck: Advisor/Consultant **Frank Tverdek, PharmD**, Merck: Advisor/Consultant **Michael J. Boeckh, MD PhD**, Allovir: Advisor/Consultant|Allovir: Grant/Research Support|AstraZeneca: Advisor/Consultant|AstraZeneca: Grant/Research Support|Merck: Advisor/Consultant|Merck: Grant/Research Support|Moderna: Advisor/Consultant|Moderna: Grant/Research Support|Symbio: Advisor/Consultant **Steven A. Pergam, MD, MPH**, Cidara: Advisor/Consultant|F2G: Advisor/Consultant|Global Life Technologies: Grant/Research Support|Symbio: Advisor/Consultant **Catherine Liu, MD**, Pfizer: Grant/Research Support

